# Invertebrate and avian predators as drivers of chemical defensive strategies in tenthredinid sawflies

**DOI:** 10.1186/1471-2148-13-198

**Published:** 2013-09-16

**Authors:** Jean-Luc Boevé, Stephan M Blank, Gert Meijer, Tommi Nyman

**Affiliations:** 1 Department of Entomology, Royal Belgian Institute of Natural Sciences, Rue Vautier 29, B-1000 Brussels, Belgium; 2 Senckenberg Deutsches Entomologisches Institut, Eberswalder Straße 90, D-15374 Müncheberg, Germany; 3 Ninovestraat 6, B-9450 Haaltert, Belgium; 4 Department of Biology, University of Eastern Finland, P.O. Box 111, FI-80101 Joensuu, Finland; 5 Institute for Systematic Botany, University of Zurich, Zollikerstrasse 107, CH-8008 Zurich, Switzerland

**Keywords:** Insects, Tenthredinidae larvae, Visual signals, Deterrent hemolymph, Volatiles, Chemical defense, Predatory birds and ants, Predator–prey interactions, Diversity, Evolution

## Abstract

**Background:**

Many insects are chemically defended against predatory vertebrates and invertebrates. Nevertheless, our understanding of the evolution and diversity of insect defenses remains limited, since most studies have focused on visual signaling of defenses against birds, thereby implicitly underestimating the impact of insectivorous insects. In the larvae of sawflies in the family Tenthredinidae (Hymenoptera), which feed on various plants and show diverse lifestyles, two distinct defensive strategies are found: easy bleeding of deterrent hemolymph, and emission of volatiles by ventral glands. Here, we used phylogenetic information to identify phylogenetic correlations among various ecological and defensive traits in order to estimate the relative importance of avian *versus* invertebrate predation.

**Results:**

The mapping of 12 ecological and defensive traits on phylogenetic trees inferred from DNA sequences reveals the discrete distribution of easy bleeding that occurs, among others, in the genus *Athalia* and the tribe Phymatocerini. By contrast, occurrence of ventral glands is restricted to the monophyletic subfamily Nematinae, which are never easy bleeders. Both strategies are especially effective towards insectivorous insects such as ants, while only Nematinae species are frequently brightly colored and truly gregarious. Among ten tests of phylogenetic correlation between traits, only a few are significant. None of these involves morphological traits enhancing visual signals, but easy bleeding is associated with the absence of defensive body movements and with toxins occurring in the host plant. Easy bleeding functions through a combination of attributes, which is corroborated by an independent contrasts test indicating a statistically significant negative correlation between species-level integument mechanical resistance and hemolymph feeding deterrence against ants.

**Conclusions:**

Our analyses evidence a repeated occurrence of easy bleeding, and no phylogenetic correlation including specific visual signals is significant. We conclude that the evolution of chemically-based defenses in tenthredinids may have been driven by invertebrate as much as by avian predation. The clear-cut visual signaling often encountered in the Nematinae would be linked to differential trends of habitat use by prey and predators. Further studies on (prey) insect groups should include visual signals and other traits, as well as several groups of natural enemies, to better interpret their relative significance and to refine our understanding of insect chemical defenses.

## Background

Insects live under the Sword of Damocles, since numerous vertebrate and invertebrate predators attempt to eat them [1,2]. Predation is therefore a primary driving force in the evolution of insects, which survive biotic attacks among others by chemically based defense strategies, and an intriguing interspecific diversity in defense strategies is observed (*e.g*., [3-5]). A specific defense strategy varies during ontogeny, and relates to an adapted phenology, behavior, morphology, physiology, and/or chemistry [6,7]. Defense strategies of living organisms are shaped by evolutionary conservatism and ecological factors, but few studies have attempted to estimate the relative importance of each of these two influences by a large-scale analysis of a given insect group [8-11]. This is understandable, since ‘eco-evo’ processes of systems including insect prey and their predators are intrinsically complex [12]. We emphasize here three major points contributing to this complexity.

**Figure 1:**
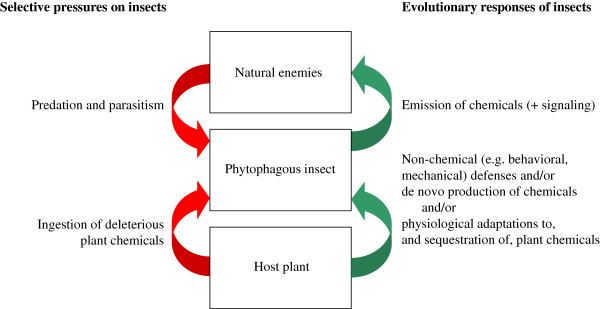
**Evolutionary interactions among trophic levels influencing chemical defensive strategies in phytophagous insects.** Phytophagous insects are held in ‘ecological pincers’ consisting of top–down as well as bottom–up selective pressures in the case of host plants containing deleterious chemicals (*red arrows*). However, the insects may sequester plant compounds, and/or produce defensive chemicals themselves, and they can also combine chemical with non-chemical defensive traits, which are all traits eventually used upon attack by natural enemies (*green arrows*).

First, numerous insects are herbivorous, which gives them the possibility to reallocate toxic or harmful plant compounds to their own benefit (Figure 1). Sequestration is the uptake and accumulation of exogenous allelochemicals in specific organs [13], but other possible fates of plant allelochemicals are, for example, their detoxification or excretion by the insect [14]. Further, defense chemicals can be produced endogenously [15]; such *de novo* production can occur in non-herbivores, but surprisingly also in herbivores feeding on plants containing deleterious allelochemicals. Species may benefit from this by becoming more independent from the plant, and by combining exo- and endogenous production, insects can facilitate their shifts to novel host-plant species [10,16,17].

Second, numerous insects prey on other insects, and such species exhibit fundamental differences in their hunting strategy as compared to insectivorous vertebrates. Even though some predatory insects are visual hunters, most tend to locate and identify potential prey primarily by means of olfactory and gustatory cues [18,19]. This contrasts with vertebrate predators such as birds, which almost exclusively rely on vision when foraging [20-23], even if tasting is an important second step [24]. The point is that we perceive our environment as birds do, prevalently by sight, which may explain why many studies focus on visual signals such as crypsis, aposematism and its often associated traits, gregariousness and mimicry. Thus, ecological factors determining the evolution of chemical defenses in insects are less studied than the signaling of such defenses [25] (Figure 1).

Third, defensive chemicals are often multifunctional. Bioactive compounds can be general irritants acting on the peripheral sensory system, or toxins of specific physiological action [26]. Chemically, they roughly correspond to volatiles and water-soluble compounds, respectively. An advantage (for the emitter) of volatiles is that they keep the predator at a distance, whereas the action of water-soluble compounds requires ingestion or at least contact by the predator; repellence is defined here as involving the olfactory system, whereas feeding deterrence the gustatory one [27]. However, all such chemical and functional distinctions remain quite arbitrary. Defensive chemicals in one species are often a mixture of chemicals and can be multifunctional by including chemical precursors, solvents, and/or wetting agents of the active compounds, by showing a feeding deterrence and toxicity, or a repellent and topical activity, etc. [4,5,15,28-31]. Even a single compound can be multifunctional [32], and different compounds often act in synergy [33]. More generally, dose-dependent effects of a chemical are ubiquitous, as already observed about 500 years ago by Paracelsus (*e.g.*, [34-36]). Finally, the interspecific activity of allelochemicals have led to a subset of names and definitions depending on the beneficial/detrimental action of the compounds for the emitter *versus* receiver, but again, a given compound can fulfill several of such ecological functions [37].

To better understand the evolution of chemical defensive strategies in phytophagous insects, we aimed to reconstruct the phylogeny of the Tenthredinidae sawflies, which constitute the major group of herbivorous Hymenoptera, and which show a large diversity in life histories. Tenthredinids exhibit high intimacy with their host plant since females lay their eggs into the plant tissue [11]. Their larvae generally live freely on plant leaves and are preyed upon by numerous vertebrate and invertebrate predators [38]. Two distinct chemical defensive strategies are known among tenthredinid larvae. On the one hand, species in the subfamily Nematinae possess eversible ventral glands, which emit a volatile secretion that is probably aimed primarily against predatory insects and secondarily towards birds [39]. On the other hand, some tenthredinid species, especially those belonging to the blennocampine tribe Phymatocerini, are characterized by being able of ‘easy bleeding’, which is a phenomenon so far unknown from other insects and that is different from reflex bleeding [40]. In species able of easy bleeding, the larval integument readily disrupts under exogenous mechanical stress at any point of the body [40-42], and the oozing hemolymph that contains sequestered plant secondary metabolites [14,43-45] is strongly feeding deterrent to biting predators such as ants and wasps [40,43,46]. Comparative bioassays and modeling of the integument surface structure indicate that easy bleeders are more effectively defended against such invertebrate predators than against birds [41,47]. Besides ventral glands and easy bleeding, alternative or complementary larval defenses include a developed pubescence, an integumental secretion layer [48,49], and an endophytic lifestyle by galling, rolling, mining or boring in different plant tissues [50,51]. Moreover, there is diversity in the cryptic or aposematic appearance, and level of gregariousness among tenthredinid larvae [39,52,53]. Such a large and diversified range of defensive devices within this insect group prompted us to search for evolutionary patterns, by seeking an explanatory framework of ecological factors that would account for this diversity. Therefore, we mapped ecological and defensive traits on phylogenetic trees, and tested correlations between character pairs, with the aim to infer the relative impact of invertebrates *versus* vertebrates in the evolution of chemically-based defenses. Our general hypothesis was that if vertebrates would be the main driver in this evolution, then at least some specific visual signals should be correlated, at a large phylogenetic scale, with an underlying chemical defense (see Figure 1).

## Methods

### Study group and taxon sampling

Tenthredinidae is the largest sawfly family with over 5,500 species described worldwide, covering all continents except Antarctica [54]. Most tenthredinid species are dietary specialists: larvae typically feed on one or a few related plant genera [55]. The majority of the hosts belong to di- and monocotyledonous angiosperms, but numerous species feed on gymnosperms, ferns, horsetails, and even mosses [55]. Generally, tenthredinids have been subdivided in seven, but more recently in the six subfamilies Allantinae, Blennocampinae, Heterarthrinae, Nematinae, Selandriinae, and Tenthredininae [54].

The sawfly species used in this study comprise 106 tenthredinid species (Additional file 1), with representatives from all subfamilies and 29 of their major tribes. We primarily focused on species for which data on chemical defense traits but no accurate phylogenetic analyses were available, which is especially the case for the Phymatocerini, represented here by 22 exemplars. From 10 non-tenthredinid sawfly families, 13 species were included in as outgroups.

Sawfly adults were identified following Benson [56] and Zhelochovtsev & Zinovjev [57], and larvae with Lorenz & Kraus [48]. Specimens were stored in 100% ethanol at −20 or −80°C, and vouchers are kept at the Royal Belgian Institute of Natural Sciences (JLB collection; Additional file 1).

### DNA extraction, PCR amplification and sequencing

Total genomic DNA was extracted from legs or abdomens of adult sawflies or parts of larvae following a standard CTAB protocol. We amplified and sequenced two mitochondrial genes, Cytochrome b (Cytb) and Cytochrome oxidase I (COI), and the nuclear 28S ribosomal gene. Cytb was amplified (and the PCR products sequenced in both directions) using primers CB-J-10933 and CB-N-11367 [58]. For CoI, we used a modified version of the Simon *et al.*[58] primer C1-J-1718 (5’-GGA GGA TTT GGA AAT TGA TTA ATT CC-3’) in combination with the reverse primer mod-A2590 (5’-ACT GCT CCT ATT GAT AAT ACA TAA TG-3’; GM, own design). For 28S, the primers 28SF2 (5’-CAC GAG CCG ATA GCG AAC AAG T-3’; GM, own design) and 28SB2 (5’-CCA AGG CCT CTA ATC ATT CGC T-3’; GM, own design) were used. PCR reactions contained 10 mM Tris–HCl, 50 mM KCl, 1.5 mM MgCl_2_, 50 μM of each dNTP, 0.4 μM of each primer, and 0.026 units/μl of *Taq* polymerase (Amersham Bioscience).

The PCR programs consisted of an initial denaturation step at 94°C for 4 min, followed by 30 cycles of 94°C for 60s, annealing at 52°C (Cytb and COI) or 54°C (28S) for 60s, and extension at 72°C for 2 min. The cycles were followed by a final extension step at 72°C for 10 min. PCR products were purified using either alcohol precipitation or the Amersham Bioscience GFX PCR and Gel Band Purification Kit.

Cytb was sequenced on an Amersham ALF express automatic sequencer using Cy5 labelled primers and the Amersham sequencing kit, and with an annealing temperature of 50°C. COI and 28S products were sequenced using ABI PRISM BigDye Terminator cycle sequencing kits and a Perkin Elmer ABI sequencer at the Vlaams Instituut voor Biotechnologie in Antwerp, Belgium.

Sequences were assembled and checked using the base-calling software of the respective sequencers, and then aligned using ClustalX v. 1.81 [59]. The alignments were corrected by eye. Alignment was straightforward for the mitochondrial genes, which contained very few insertions or deletions (indels), and in which codons could serve as reference. By contrast, numerous indels were present in the 28S sequences, although these tended to occur mainly between outgroup and ingroup taxa. The final dataset contains 397, 862, and 999 aligned base pairs for Cytb, COI, and 28S, respectively (2,258 bp in all) (Additional file 2). All sequences have been submitted to GenBank under accession numbers KF528387–KF528662, and the full dataset (as well as resultant trees) are also available in TreeBase at http://purl.org/phylo/treebase/phylows/study/TB2:S14547.

### Phylogeny reconstruction

**Figure 2:**
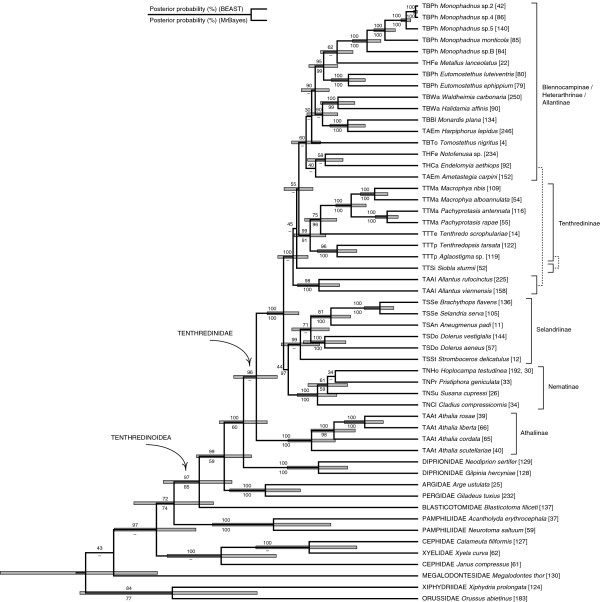
**Relaxed molecular-clock phylogeny of the Tenthredinidae and selected outgroup taxa.** The BEAST MCC tree is based on analysis of Dataset 1, which includes tenthredinids that have sequences from all three genes, and all outgroups. Numbers above branches are posterior probabilities (%) from the BEAST analysis, numbers below branches show corresponding values from the MrBayes run (clades not present in the MrBayes tree are indicated by hyphens). Grey shaded bars show 95% highest posterior density intervals for relative node ages for nodes with posterior probabilities over 50%.

**Figure 3:**
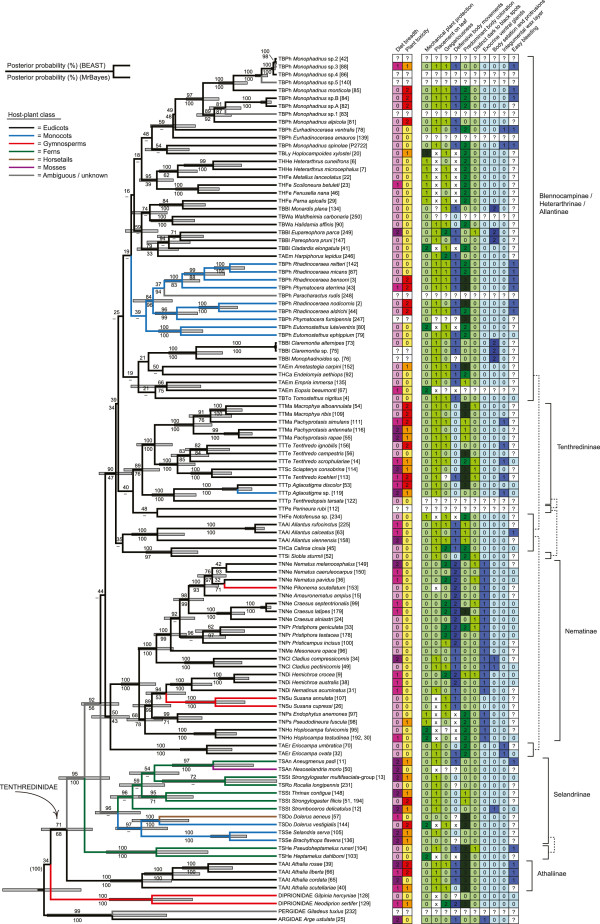
**Relaxed molecular-clock phylogeny of the Tenthredinidae, and the distribution of various larval ecological and defensive traits within the group.** The BEAST MCC tree is based on analysis of Dataset 2, which includes all sequenced tenthredinids as well as representatives from three non-blasticotomid families in Tenthredinoidea. Posterior probabilities (%) resulting from analyses in BEAST and MrBayes are given above and below branches, respectively (clades not present in the MrBayes tree are indicated by hyphens). Grey shaded bars show the 95% highest posterior density intervals for relative node ages for nodes with posterior probabilities exceeding 50%. Branch colors denote host plant classes of the sawfly species (see legend) and ancestral reconstructions based on maximum-likelihood optimization across 1,000 post-burnin trees (see Additional file 4A). In the table to the right of the tree, diet breadth, plant toxicity, and defensive traits (from left to right) are coded as shown in Table 1. (?) Unknown; (x) not applicable.

To reduce the effects of missing data, the full sequence alignment was split into two separate datasets: “Dataset 1” included all 13 outgroup taxa and the 40 tenthredinid species that had sequences of all three genes (see Figure 2). “Dataset 2” included only outgroups from non-blasticotomid Tenthredinoidea (4 spp. representing Argidae, Pergidae, and Diprionidae), and all 106 ingroup taxa (see Figure 3).

Both datasets were analyzed using Bayesian phylogenetic inference as implemented in MrBayes v. 3.1.2 [60] and BEAST v. 1.5.2 [61]. Prior to the runs, best-fitting substitution models for COI (TVM+I+G), Cytb (TVM+I+G), and 28S (GTR+I+G) were identified under the Akaike information criterion in jModelTest 2.1.3. [62]. Because the TVM model is not implemented in MrBayes v. 3.1.2, we used a separate, unlinked GTR+I+G model of substitution for each gene in all phylogenetic analyses.

The MrBayes analysis of Dataset 1 implemented default priors and included two independent runs of four incrementally heated chains (t = 0.2) that were run for 6 million generations, while sampling trees from the current cold chain once every 100 generations. The first 10,001 trees sampled prior to chain stationarity were discarded as a burnin from each run, and a Bayesian consensus tree showing all compatible groupings was calculated on the basis of the 100,000 trees that remained in the combined tree sample. Runs with Dataset 2 were otherwise similar, but each run included six chains with the temperature parameter set to 0.1, and the analysis was run for 10 million generations. After deleting a burnin of 30,001 trees from both runs, a consensus tree was calculated on the basis of the remaining 140,000 trees.

Topologically unconstrained BEAST runs of Dataset 1 employed an uncorrelated relaxed lognormal clock model of rate variation among branches, a Yule prior on speciation, and default priors for other parameters. Two independent runs with automatic tuning of operators were run for 60 million generations, while sampling trees and parameters every 1,000 generations. After discarding 10,001 trees from both runs as a burnin, the tree files were combined using LogCombiner (part of the BEAST package). A maximum clade credibility (MCC) tree showing mean node heights was then calculated on the basis of the 100,000 post-stationarity trees in TreeAnnotator (part of the BEAST package). Settings in the runs involving Dataset 2 were mostly identical (Additional file 3), but we ran four independent analyses from which trees were combined after a burnin of 10,001 trees. The combined tree file was then thinned by resampling trees every 4,000 generations, and an MCC tree was calculated based on the remaining 50,000 post-stationarity trees.

### Character coding and reconstruction of ancestral states

In order to infer the evolutionary history of traits related to niche use and defense within Tenthredinidae, we collected data on diet breadths, host-plant associations, and host features, as well as on larval ecology, behavior, morphology, and chemically-based defensive strategies in the species included in the phylogenetic trees. We then coded the data as unordered binary or multistate characters, and reconstructed ancestral states by single-rate (Mk1) maximum likelihood (ML) optimization in Mesquite v. 2.75 [63]. To accommodate phylogenetic uncertainty, reconstructions were performed across 1,000 post-burnin trees obtained by resampling trees from the Dataset 2 BEAST tree sample at regular intervals in LogCombiner, and results were summarized across the nodes of the MCC tree (Figure 3) from the same analysis (Additional file 4). ML optimization in Mesquite is not possible if taxa with unknown or polymorphic states are present in the focal character, so such species were deleted from the matrix and associated trees prior to each reconstruction.

Host plants were compiled from literature sources [55,64] and each sawfly was coded for its diet breadth (Figure 3). In the case of species for which reliable host-plant records were missing, diet breadth was coded as unknown, unless revealed by further laboratory testing with larvae from the same population.

To evaluate the toxicity of plants on which sawflies feed, each plant genus (and species, as far as possible) was associated with the occurrence of toxins, by referring to standard works on the chemistry of plants [65-70] and to smaller and/or more recent works (*e.g.*, [45]). A plant taxon was considered toxic if the leaves contain secondary metabolites from one or more of the following chemical classes: alkaloids (including steroid alkaloids and steroid saponins, which are closely related in terms of biosynthesis and metabolism; [71]), glucosinolates, cyanogenic glycosides, and non-protein amino acids. All these compounds, except steroid saponins, have in common the inclusion of one or more nitrogen atoms. Nitrogen-containing secondary metabolites show acute toxicity and/or strong feeding deterrence towards vertebrates and/or invertebrates, and they are the most common defensive chemicals of plants [65,68-70,72]. A plant taxon was considered non-toxic if it only contains secondary metabolites that do not contain nitrogen, such as phenolics (*e.g.*, coumarins, phenolic glycosides, and the widespread flavonoids), terpenoids (*e.g.*, iridoid glycosides, triterpenoid saponins), or ranunculin (characteristic of the Ranunculaceae). Following the specific host plant(s) of each sawfly species, host toxicity was then coded as ‘never’ (code ‘0’), sometimes (‘1’), or ‘always’ (‘2’), depending on the possible occurrence of toxins in the diet. For instance, the code was ‘0’ for a specialist sawfly species feeding on a non-toxic plant genus, ‘1’ for a generalist feeding on both toxic and non-toxic hosts, and ‘2’ for a sawfly species only feeding on a toxic plant, or feeding on several plant taxa which are all toxic.

Ten ecological traits linked to the behavior, morphology and chemical ecology of the sawfly larvae were coded as far as these traits are involved in defense (see Figure 3). The data were extracted from standard works on sawflies (*e.g.*, [48,55,64,73] and literature therein), a specific work on easy bleeding [40], as well as unpublished observations and sources. For traits changing during successive larval stages, the last stage preceding the (often non-feeding) eonymph was considered.

### Correlation analyses

The existence of phylogenetic correlations among various ecological and defensive traits was evaluated by Bayesian stochastic character mapping [74,75] as implemented in SIMMAP v. 1.5.2 [76]. For these analyses, we selected 10 out of the 66 character-pair comparisons that are possible among the 12 focal traits listed in Table 1. Most correlations to be performed were selected based on previously proposed hypotheses (see [39,40,47] and Table 2). State-by-state associations between characters were evaluated based on the *d*_*ij*_ statistic, which measures co-occurrence of states *i* and *j* across branches in relation to the expectation under independent evolution [75]. Overall character correlations were measured using statistic *D*, which is the sum of the absolute values of individual *d*_*ij*_’s between characters [75].

**Table 1:** Plant features plus ecological and defensive traits of tenthredinid sawfly larvae used in reconstructing ancestral states and analyzing phylogenetic correlations

**Character**	**(Code) state**
Diet breadth	(0) one plant species or genus, (1) at least two plant genera but of one family, (2) plant genera of at least two families
Plant toxicity	(0) never, (1) sometimes, (2) always
Mechanical plant protection	(0) free-living larva, (1) leaf miner, (2) borer, (3) galler
Placement on leaf	(0) leaf edge, (1) leaf upper- and/or underside
Gregariousness	(0) solitary, (1) aggregated, *i.e.*, larvae distributed on a plant, generally < 3 per leaf, (2) truly gregarious, *i.e.*, larvae on one leaf or several adjacent leaves
Defensive body movements	(0) dropping easily and/or violent movements, (1) no, (2) raising abdomen
Predominant body coloration	(0) green, (1) white ventrally and green dorsally, (2) white or yellow, (3) brown-grey to black, or white ventrally and dark dorsally
Distinct dark to black spots	(0) absent, (1) present
Exocrine ventral glands	(0) absent, (1) present
Body setation and protrusions	(0) with very short setae and without long protrusions, (1) with setae > 1/6 as long as body diameter, (2) with protrusions or spines > 1/6 as long as body diameter
Integumental wax layer	(0) no, (1) yes
Easy bleeding	(0) no, (1) yes

**Table 2:** **Overall phylogenetic correlations between various ecological and defensive characters (*D*) and associated *P*-values, estimated by Bayesian stochastic mapping across a sample of 500 post-burnin trees**

**Ref.**	**Character (code)**	**Character (code)**	***D***	***P***
[40]	Diet breadth (1)	Plant toxicity (2)	0.196	0.010
	Plant toxicity (2)	Mechanical plant protection (3)	0.104	0.056
[40]	*Plant toxicity (2)*	*Easy bleeding (12)*	0.260	**0.000**
	Placement on leaf (4)	Integumental wax layer (11)	0.032	0.198
[39]	Gregariousness (5)	Defensive body movements (6)	0.230	**0.000**
[39]	Gregariousness (5)	Dark spots on body (8)	0.061	0.468
[39]	Defensive body movements (6)	Dark spots on body (8)	0.078	0.164
[47]	*Defensive body movements (6)*	*Easy bleeding (12)*	0.444	**0.004**
	Predominant body coloration (7)	Body setation and protrusions (10)	0.113	0.048
[40]	*Predominant body coloration (7)*	*Easy bleeding (12)*	0.109	0.024

Most comparisons were selected by following the mentioned references (Ref.). Results from character-by-character analyses performed using a reduced dataset are given in italics, and *P*-values remaining statistically significant (at *P* < 0.05) after Holm's sequential Bonferroni correction are given in bold. See Additional file 5 for detailed results.

Before the main analyses, parameter priors were determined based on MCMC analyses following the approach of Schultz and Churchill [77] in SIMMAP. For binary characters, best-fitting priors for the bias parameter (= beta distribution parameter α) and the overall evolutionary rate parameters (= gamma distribution parameters α and β), were determined based on the Dataset 2 BEAST MCC tree (Figure 3), which had been rescaled to a length of 1. MCMC runs were performed using the default number of distribution discretization categories (31 for the bias parameter and 60 for the rate parameters), cycles (100,000), sampling frequency (200), burnin (10,000), and upper rate bound (1,000). Results were extracted in R v. 2.14.0 [78] using the sumprmcmc.r script provided in the SIMMAP installation package. In the case of multistate characters, we used an empirical prior for the bias parameter, while rate-parameter priors were determined as described for binary characters. All characters were treated as unordered.

In order to accommodate phylogenetic uncertainty, all correlation analyses were performed across 500 post-burnin trees (rescaled to a length of 1) obtained by regularly thinning the original Bayesian tree sample from the Dataset 2 BEAST runs in LogCombiner. Pairwise correlation analyses were configured using the aforementioned bias and rate priors for each character. The number of samples, prior draws, and predictive samples were set to 1, meaning that both observed and predictive sample sizes equaled 500 for each character pair. For the three pairwise tests involving character 12 (= easy bleeding), the high amount of missing data in this trait led to exceedingly long run times. Therefore, these analyses were instead based on a reduced dataset, in which the tree used for prior determination, as well as the ones in the 500-tree sample, were pruned to include only those taxa that had a known state for easy bleeding.

### Independent contrasts test

**Figure 4:**
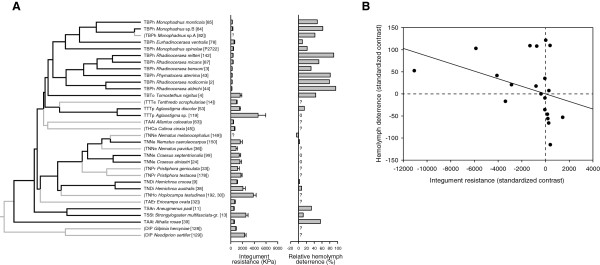
**Part of the phylogenetic tree of tenthredinids with estimated levels of traits linked to easy bleeding, and plot of independent contrasts extracted from a phylogeny that includes only species with no missing data.** The tree in **(A)** was obtained by pruning the BEAST MCC tree in Figure 3, plots on the right-hand side of the tree show levels of integument resistance and hemolymph deterrence estimated for the included species ([40,41] and U. Schaffner, unpublished data). Species excluded from the independent contrasts test due to missing data are denoted by gray terminal branches and parenthesized names. The scatterplot in **(B)** shows standardized contrasts for 21 nodes on the tree that include only species that have estimates for both traits, as well as the regression line forced through the origin.

In a survey of nine species in the blennocampine tribe Phymatocerini, Boevé & Schaffner [40] found a statistically highly significant negative correlation between integument resistance and hemolymph deterrence, that is, the less the integument is resistant to a standardized mechanical stress, the more ants are deterred by the hemolymph, and vice versa. However, their analysis treated species as independent data points, which can potentially lead to spurious results due to phylogenetic non-independence of species [79]. Hence, we re-analyzed an expanded dataset using Felsenstein’s [80] independent contrasts method implemented in the PDAP:PDTREE package v. 1.15 [81] in Mesquite. These expanded analyses were based on 21 tenthredinid species for which both integument resistance and hemolymph deterrence had been measured [40]. The tree used in these analyses (a reduced version of the one shown in Figure 4A) was obtained by pruning the BEAST MCC tree in Figure 3.

## Results

### Phylogenetic trees

The trees from the sequence data reveal evidence for the monophyly of the Tenthredinidae (Figures 2 and 3), as indicated earlier [82]. Outside Tenthredinoidea, however, missing data in some outgroup representatives lead to clearly wrong groupings in Dataset 1 analyses, so the basal parts of the tree (Figure 2) should be treated with caution. This particularly concerns the placement of Xyelidae within Cephidae in the BEAST MCC tree, as well as the apparent polyphyly of the Pamphilioidea (Pamphiliidae + Megalodontesidae) in both analyses (*cf*., *e.g.*, [83]). Within Tenthredinidae, the tree topologies are congruent in the monophyly and basal positioning of the genus *Athalia*, which justifies its placement in a distinct subfamily, the Athaliinae, as proposed earlier (*e.g.*, [55]). The topologies are also congruent in confirming the monophyly of the Nematinae [82]. Representatives of the Selandriinae, with the exception of the tribe Heptamelini, are grouped together. Each of the remaining traditional subfamilies, *i.e.*, the Allantinae (with the aforementioned exclusion of *Athalia*), Blennocampinae, Heterarthrinae, and Tenthredininae, come out as polyphyletic, and the groups are generally supported by low posterior probabilities. In traditional classifications, the Allantinae was, indeed, recognized very soon as an arbitrary group [84], which is less the case for the three other subfamilies. However, in all subfamilies higher probabilities are obtained at lower-level (younger) clades, which allows the following conclusions.

Easy bleeding is particularly common among a Blennocampinae tribe, the Phymatocerini ([40], Figure 3), which is a group defined by a combination of morphological characters [73]. Our analysis does not demonstrate its monophyly (Figure 3) and rather shows two distantly related clades, one ‘centered’ on *Monophadnus*, and another on *Rhadinoceraea.* The latter clade includes *Phymatocera* and *Paracharactus*, and *Eutomostethus* is close to it. The weakly supported exclusion of *Monophadnus spinolae* from other *Monophadnus* species, as well as the strong support for the grouping of *Rhadinoceraea* + *Phymatocera* + *Paracharactus*, are both reflected by morphological characters ([73], SMB, personal observation). The fact that the Phymatocerini are unique among the Blennocampinae in commonly feeding on plants containing steroidal saponins and alkaloids [40], which is clearly not a trait considered in the traditional classification of sawflies, lends additional support to the hypothetical monophyly of this tribe.

### Defense diversity

A large diversity of lifestyles and defensive traits is found in tenthredinid larvae (Figure 3). Some traits evolved repeatedly, in at least two species groups, such as easy bleeding in *Athalia* and the Phymatocerini, leaf mining in the (possibly polyphyletic) Heterarthrini and Pseudodineurini, and an integumental wax layer in some Blennocampinae and Tenthredininae, and Allantinae (Additional file 4). In contrast, other traits are known from only one taxon. Examples are the eversible ventral glands in the Nematinae, the slimy covering in *Caliroa*, hemolymph spitting in *Siobla*, and fruit boring in *Hoplocampa* (Additional file 4). Moreover, a single species can combine at least two traits, for instance, aposematism and gregariousness, crypsis and a solitary lifestyle, the presence of ventral glands and an endophytic lifestyle, or ventral glands and aposematism. However, easy bleeding and the presence of ventral glands never co-occur, meaning that no easy bleeder possesses ventral glands, and that nematine species are never easy bleeders (Figure 3). The trees also indicate that easy bleeding appeared (and was lost) at least five times: in the Athaliinae, Allantinae, Selandriinae, Tenthredininae, and Blennocampinae (Phymatocerini), with a radiation of the phenomenon within the last of these taxa (Figure 3, Additional file 4).

The wide range in overall diet breadth of tenthredinids impedes the recognition of a clear host-affiliation pattern for sawfly subgroups on host plant families and even orders. Most tenthredinid species feed on eudicots, with the two major exceptions that most Selandriinae feed on pteridophytes or monocots, and part of the Phymatocerini feed on monocots (Additional file 4). Plants containing toxic secondary metabolites are the host for species of *Athalia*, Selandriinae, (leaf-mining) Nematinae as well as the two Phymatocerini, *Monophadnus*- and *Rhadinoceraea*-centered, clades (Figure 3, Additional file 4).

### Associations among traits

From the ten chosen pairwise comparisons, six yielded statistically significant overall correlations, but only three of them remain significant after Holm’s sequential Bonferroni correction: plant toxicity with easy bleeding, gregariousness with defensive body movements, and such movements with easy bleeding (Table 2, Additional file 5). More specifically, the results indicate that plant toxicity is associated with easy bleeding, easy bleeding with the absence of defensive body movements, a solitary habit with dropping and/or violent movements, aggregation with the absence of defensive movements, and true gregariousness with raising abdomen (Additional file 5).

Felsenstein’s independent contrasts test revealed a statistically significant negative correlation between species-level integument resistance and the rate of hemolymph deterrence (*r* = −0.393, *r*^2^ = 0.155, *P* = 0.039; Figure 4B).

## Discussion

The description and analysis of chemical defense mechanisms across insects, mainly in lepidopteran and coleopteran herbivores, initiated the search for general trends in the taxonomic distribution and evolution of such mechanisms. Research using empirical and manipulative tests on predator–prey systems, computational modeling, and phylogeny-based approaches has identified sequential steps in the evolution of prey defensive traits as well as plant–insect interactions (*e.g*., [8,14,85-90]). However, nearly all such studies, even when they embrace multitrophic interactions at once, focus explicitly or implicitly on (dis)advantages as well as evolutionary sequences and consequences of visual prey signals. In this context, there is good evidence that the evolution of aposematism is accompanied by an increased diversification of lineages, as shown by paired sister-group comparisons in insects and other animal taxa [91]. Further, chemical adaptation (unpalatability) preceded morphological (warning coloration) and behavioral (gregariousness) adaptations in insects [8,85,87,89,92]. However, the next step in understanding the evolution and diversity of insect chemical defenses is to explain how unpalatability itself evolved, which remains a largely unexplored question.

Since distastefulness in aposematic phytophagous insects often relies on plant chemistry, dietary specialization would favor aposematism due to physiological processes needed to cope with the ingested toxins [14,93]. Chemical specialization that is not necessarily related to plants’ taxonomic affiliation also promotes aposematism, while similar chemical profiles of secondary compounds across plant taxa facilitate niche shifts by phytophagous insects [10,93,94], which in turn may enhance the diversity of chemicals underlying aposematism. But, shifts in resource or habitat are probably less common than previously expected, as shown for sawfly larvae and caterpillars [95,96], and all aforementioned considerations are true for exogenous but not endogenous insect toxins, because these are *per se* unrelated to host affiliation. By the examination of an insect group with defensive features including, among others, bright and cryptic colorations, we could evaluate the probably literature-biased prevalence of avian over invertebrate predation in the evolution of insect defensive strategies.

Our study reveals a diversity of defensive strategies. The tree-based analyses confirm previous conclusions from chemical and bioassay analyses on selected plant–insect systems. First, easy bleeding is a defense based on toxins from plants [40,43-45] since easy bleeders tend to feed on plants that contain such chemicals (Additional file 5). Second, easy bleeders move slowly and become immobile once disturbed, whereas other defensive behaviors are associated with non-easy bleeders ([47], Additional file 5). A third significant association to arise from our analyses is between defensive body movements and gregariousness. These behaviors are components of visual signals, but they impact predator–prey interactions also physically. They are effective towards birds as well as invertebrates, for instance, when attacking ants can be knocked or dislodged by defensive body movements, or when foraging ants ignore the presence of an immobile larva [39,47,97]. Associations including more specifically visual traits of the tenthredinid larvae were expected to be significant. Each of the two traits, ‘dark spots on body’ and ‘predominant body coloration’, was tested against two other traits, but, surprisingly, none of these four associations is significant (Table 2). In particular, ‘dark spots on body’, which contributes to a conspicuous coloration is not associated with gregariousness. This contradicts with studies on several insect groups, including the Nematinae, that emphasize the link between aposematic coloration and gregariousness [8,9,39,52,87,98]. It seems that by studying the vast group of the tenthredinids we incorporated multiple defensive traits related to visual, mechano-physical and/or chemical cues, without focusing only on those known *a priori* to be directed against birds.

Besides insectivorous birds, predatory insects and especially ants are known to shape communities and influence the evolution of sawflies [99,100], and besides easy bleeding, a second main defensive strategy is the presence of volatile-emitting ventral glands. Both easy bleeding and ventral glands are most effective against predatory insects such as ants, and less so against birds [39,47]. We assume intertwined roles played by invertebrate and vertebrate predators on the evolution of defensive strategies in tenthredinids. Their basal taxon, *Athalia*, and other taxa use easy bleeding as defense, and the tenthredinids has radiated into species-rich groups such as the Selandriinae (970 species), Nematinae (1,250), and Tenthredininae (1,720) [54], which illustrates the success of the family. Predation is generally believed to be a main driver in the evolution of insects, and the observed patterns suggest that the evolution and radiation of several tenthredinid subgroups have been driven by invertebrate rather than by vertebrate predators, and by which easy bleeding arose as a first defensive strategy. It remains unknown why this unique defensive strategy did not evolve in other insects while it was gained and lost several times in tenthredinids (Figure 3 and Additional file 4). Conversely, the use of a volatile secretion produced by exocrine glands is rather common in insects [4], but within the tenthredinids it is restricted to the Nematinae, and defense by ventral glands therefore seems to be an alternative to easy bleeding. To be effective, the two defense strategies require quite opposite behaviors, by raising the abdomen and by becoming immobile, respectively, which may explain why they evolved in a mutually exclusive way. In contrast, more exclusive visual signals could theoretically complement both of them.

It is then intriguing that the Nematinae include relatively frequent cases of brightly colored and truly gregarious species ([48,53], Figure 3), which indicates a more specific evolutionary impact of birds. Since both easy bleeding and ventral glands are primarily directed against invertebrate predators [39-41,47], the paradox is that only the latter defense is repeatedly linked to aposematic coloration, while the chemistry underlying both defenses is potentially as effective against invertebrates as against vertebrates (see later), and volatiles are not particularly effective against birds. *Athalia*, Nematinae, and also Phymatocerini appeared within a relatively short time range or even concurrently (Figures 2 and 3), so that it is not likely that one defense strategy arose much later than the other, in response to a new predation type. The Nematinae compared to all other tenthredinids are however singular in having their greatest diversity in the northern Holarctic, and they have a propensity to feed on willows (*Salix*) and other trees and shrubs, whereas most *Athalia* feed on Brassicaceae, and most Phymatocerini on Ranunculales and Liliales [55,73,95], the three latter groups of plants being herbaceous. Insectivorous birds forage commonly in open (understory and canopy) forest habitats and probably less often at ground-level where they themselves suffer a higher predation risk [101-103], whereas ants occur more equally across all vegetation levels [104]. Differential trends of microhabitat-linked predation pressures, by ants and birds on Nematinae *versus* more prominently by ants on *Athalia* and Phymatocerini, may have driven the evolution of differing defensive traits [105]. The underlying chemicals, water-soluble compounds and volatiles, are dissimilar, too. Both types of chemicals can act on invertebrates as well as vertebrates [4,31,68,72]. Since any predator logically approaches before it attacks a potential prey, volatiles by acting at distance are more prone than water-soluble compounds to prevent an attack, but the latter compounds have a more profound physiological effect upon ingestion [26]. The effectiveness of different types of allochemicals is moreover affected by the predator–prey body size ratio and the consequently possible set of behavioral interactions between both protagonists [106,107]. It is within one type of allelochemicals that the diversity of selective pressures imposed by predators may promote preys’ chemical diversity, while the type itself of chemicals would be determined by basic, morphological and physiological features. How predators promote chemical defense diversity requires further analyses by focusing not only on birds [90] but also insectivorous insects. Tenthredinids are a singular group of prey insects due to the unique occurrence of easy bleeding, but our case study on them evidences general patterns of chemically-based prey adaptations, and it adds to our overall understanding of chemical defense diversity in insects.

## Conclusions

Contrasting selective pressures imposed by various natural enemies on insect herbivores are likely to lead to the evolution of distinct defensive syndromes that potentially can be identified based on phylogenetic correlations among multiple independent traits. In the family Tenthredinidae, a staggering diversity of defensive strategies has evolved, and our macro-evolutionary analyses uncover several cases of evolutionary non-independence among anti-predator traits. In the particular case of easy bleeding, an independent contrast test confirmed the existence of a negative phylogenetic correlation between the mechanical resistance of the integument and the hemolymph’s feeding deterrence towards ants. Since water-soluble compounds from the hemolymph of easy bleeders (*Athalia* and Phymatocerini species, among others) as well as volatiles from the ventral glands (in the Nematinae) are more prone to act as a defense against predatory insects than birds, it is likely that the obvious visual signaling often encountered in the Nematinae is caused indirectly by differential trends of habitat use by sawfly prey *versus* predator groups. Although several ecological and defensive traits were screened in tenthredinid larvae, none of those referring to specific visual signals were significantly correlated with the others. We conclude that, without neglecting the selective pressure by insectivorous birds, it seems necessary to emphasize the overall evolutionary impact of invertebrate predators on insect defensive strategies.

Several theories on plant-insect relationships account for the diversity of plant defenses [88], while extending such theories to predator–prey relationships is much rarer [86] because especially those interactions involving herbivorous prey can become extremely complex. The mechanisms of arms races between predators and dangerous prey imply coevolution rather than escalation [108]. From a predator’s perspective, coevolution and escalation differ in what selective agents are responsible: the defense of a prey, or the attack by a predator. From a tenthredinid’s perspective, the present study and others indicate that sawfly species face guilds of vertebrate and invertebrate predators, but also parasitoids and pathogens [109-113]. Chemicals conform but also differ in their bioactivity on distant taxonomic groups such as invertebrates and vertebrates. A partially variable bioactivity can ‘bridge’ the use of defensive chemicals from one target group to another, and, hence, promote chemical diversification. A possible pattern of the macroevolution of insect chemical defenses would be that allelochemicals effective on invertebrates were co-opted for their bioactivity on birds. There is a need for further research on such adaptive cascades in insects.

## Competing interests

The authors declare that they have no competing interests.

## Authors’ contributions

The study was conceived and designed by JLB and TN. JLB managed taxon sampling, compiled ecological data, and wrote main parts of the manuscript. GM conducted laboratory work and prepared the sequence alignments. TN performed the phylogenetic and statistical analyses, wrote related parts of the manuscript, and prepared most of the figures. SMB provided taxonomic information and identified specimens. All authors read and approved the manuscript.

## Supplementary Material

Additional file 1 Excel file containing collection data for the specimens used in the study. Click here for file

Additional file 2 NEXUS file containing the sequence data matrix, settings for MrBayes runs, and trees obtained from the Bayesian phylogenetic analyses in BEAST and MrBayes. Click here for file

Additional file 3 XML file used for the Dataset 2 phylogenetic analyses in BEAST. Click here for file

Additional file 4 **Reconstruction of ancestral states in host plant associations as well as ecological and defensive traits (A–M) based on maximum-likelihood optimization across a sample of 1,000 post-burnin trees from the Dataset 2 BEAST analysis.** Results are summarized across the MCC topology (Figure 3), pie charts on nodes show proportions of trees with uniquely best states with the decision threshold set to *T* = 2. Click here for file

Additional file 5 **Overall correlations between characters (*D*) and between states within characters (*d*) estimated by Bayesian stochastic mapping in SIMMAP based on a sample of 500 post-burnin trees.** Click here for file
