# Supplementary material for: Invertebrate and avian predators as drivers of chemical defensive strategies in tenthredinid sawflies
Source: BMC Evol Biol. 2013 Sep 16;13:198. doi: 10.1186/1471-2148-13-198 (PMC3848831; doi:10.1186/1471-2148-13-198)

A. Host-plant class

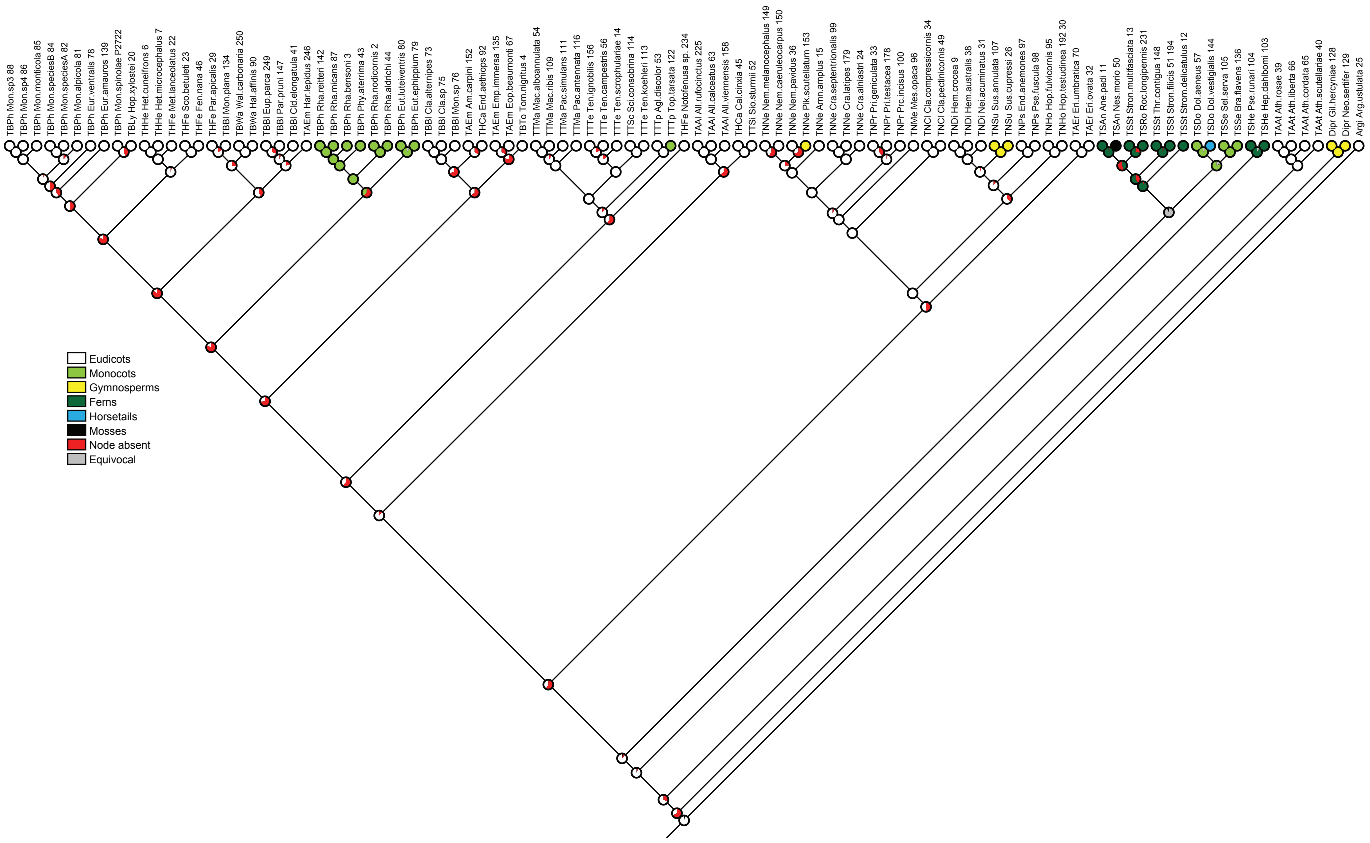

B. Diet breadth

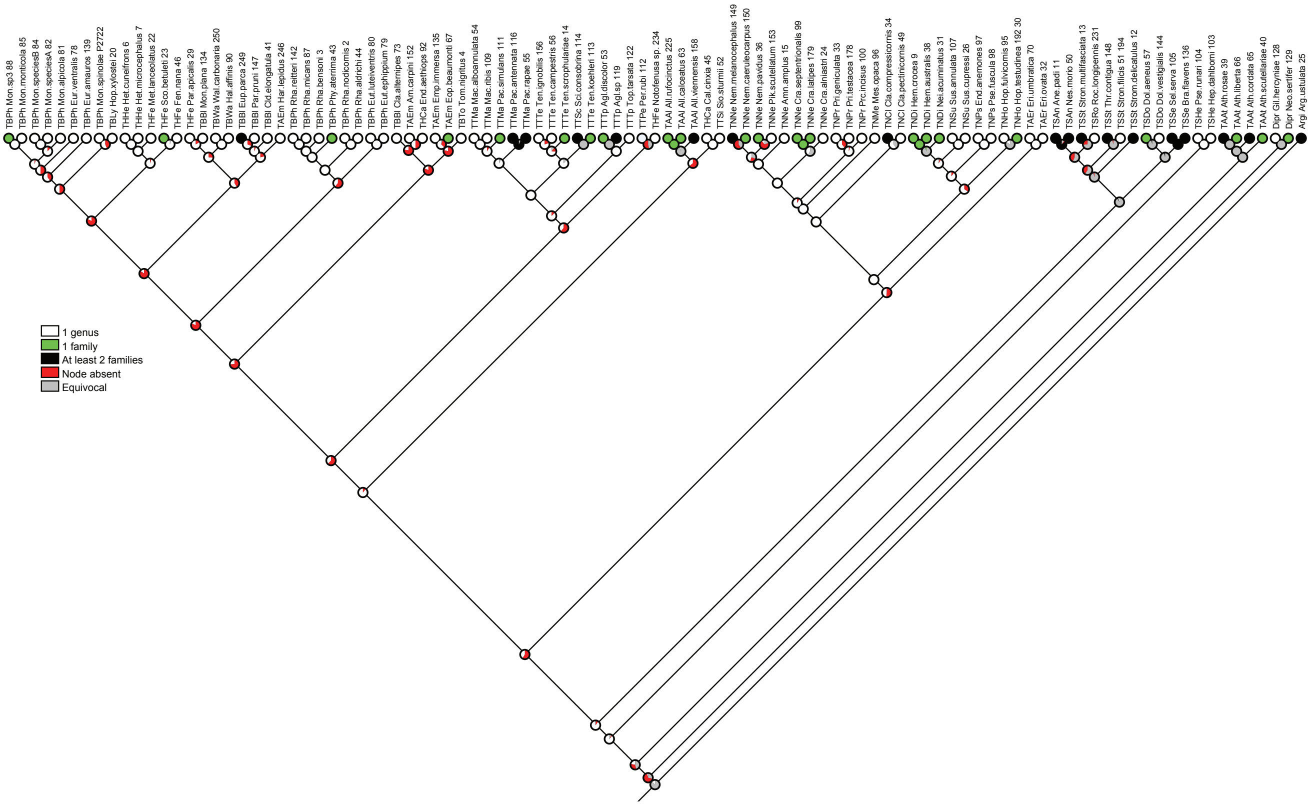

C. Plant toxicity

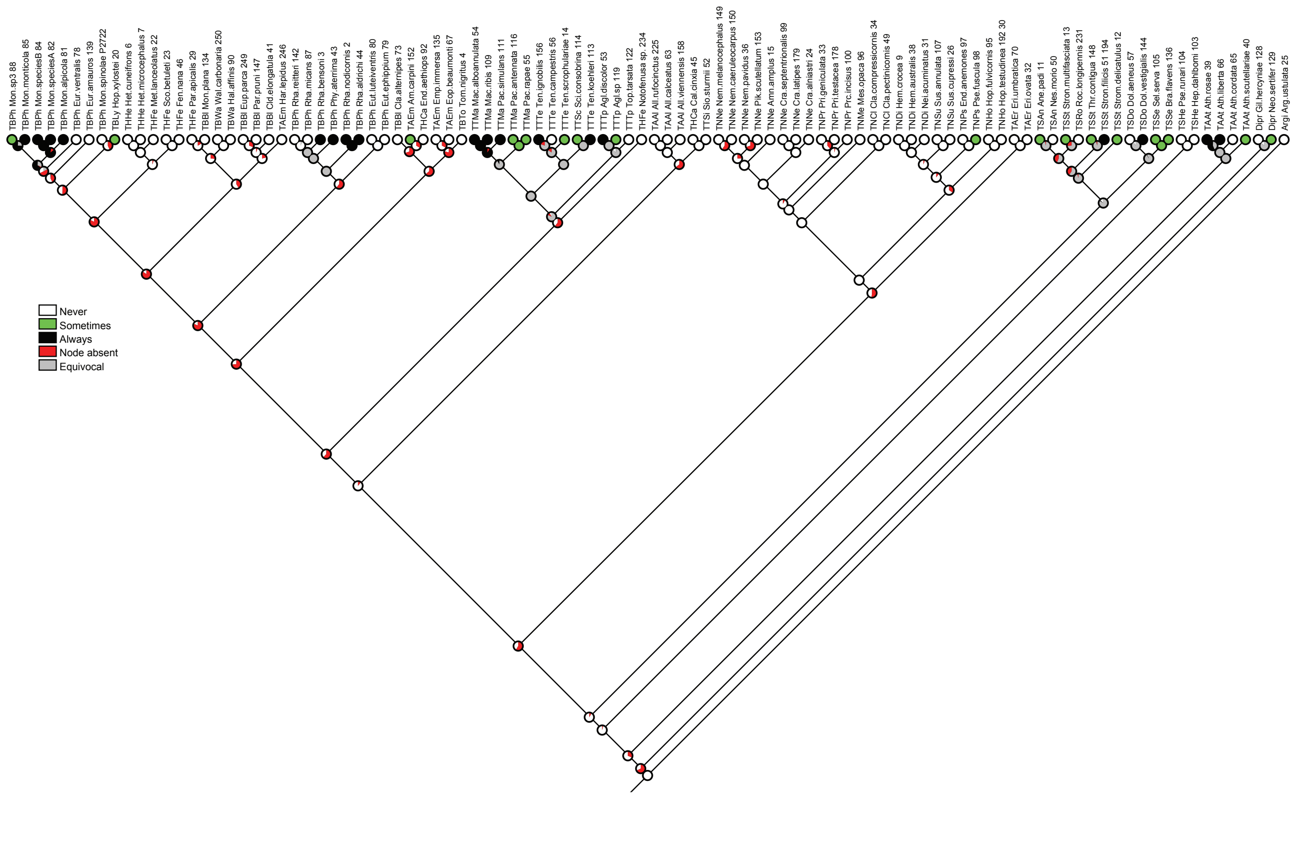

D. Mechanical plant protection

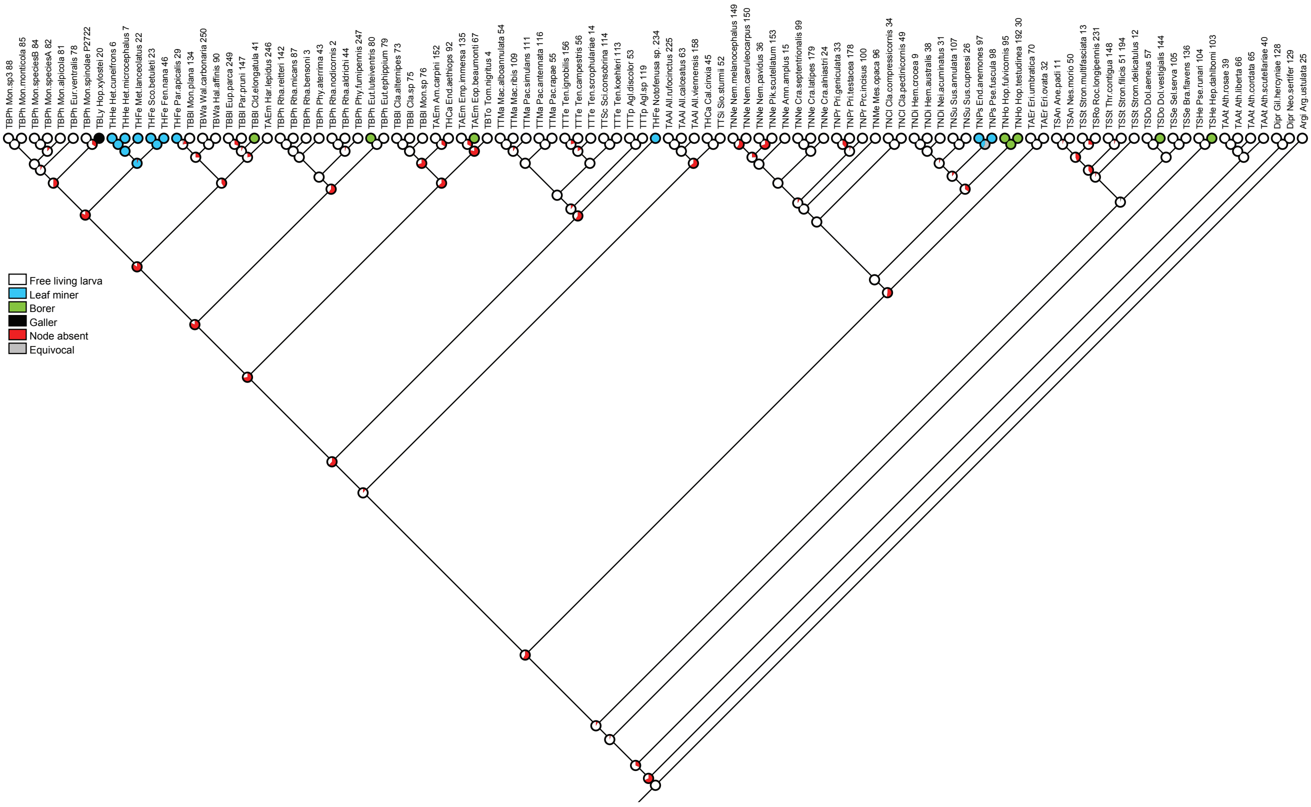

E. Placement on leaf

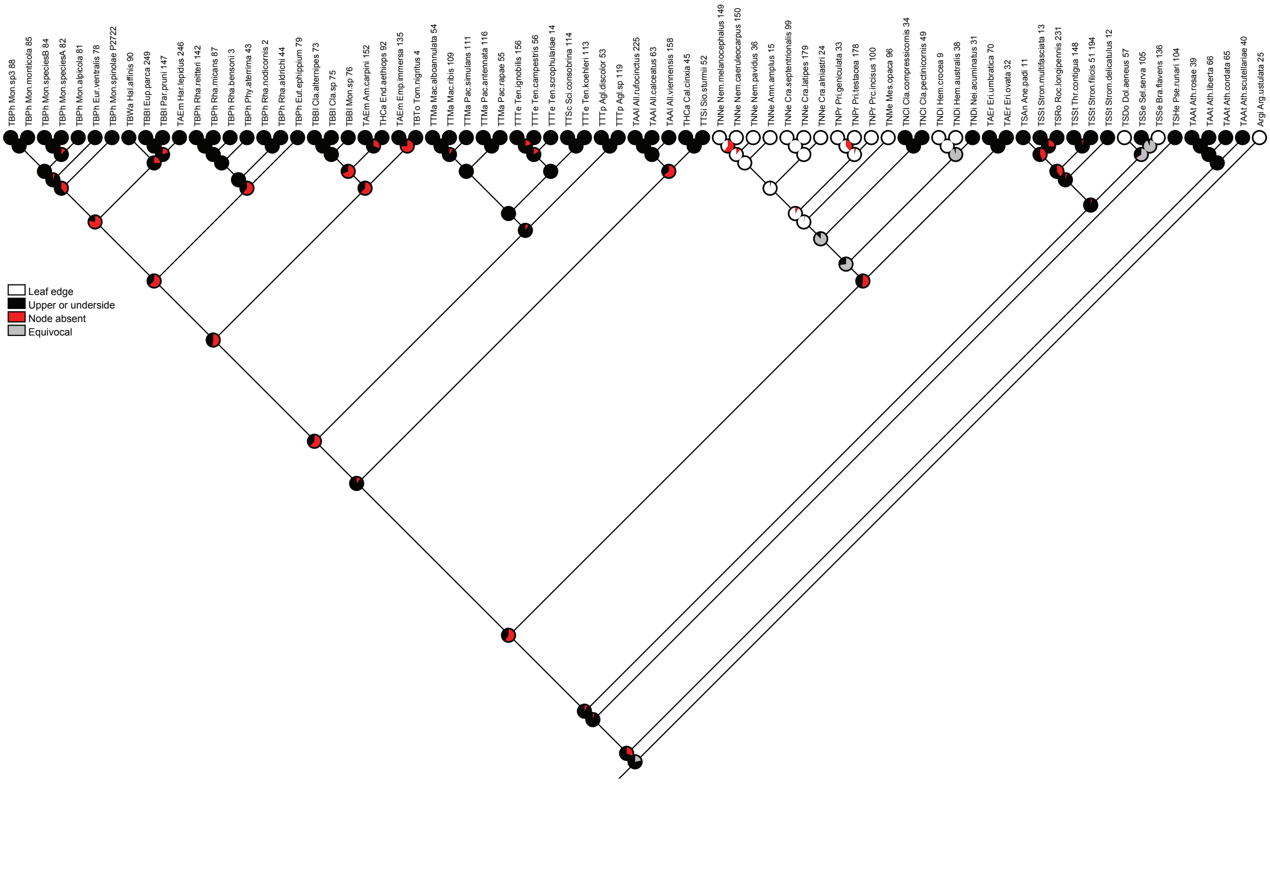

F. Gregariousness

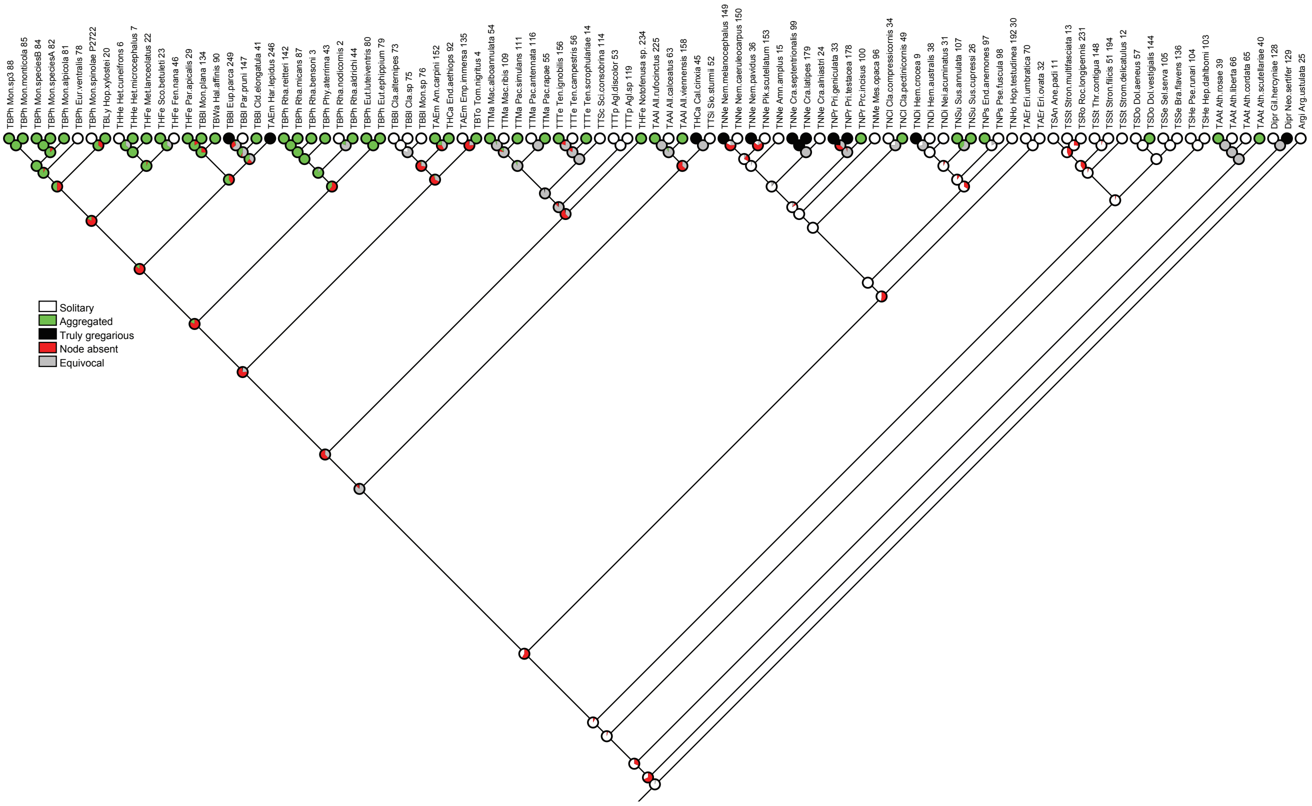

G. Defensive body movements

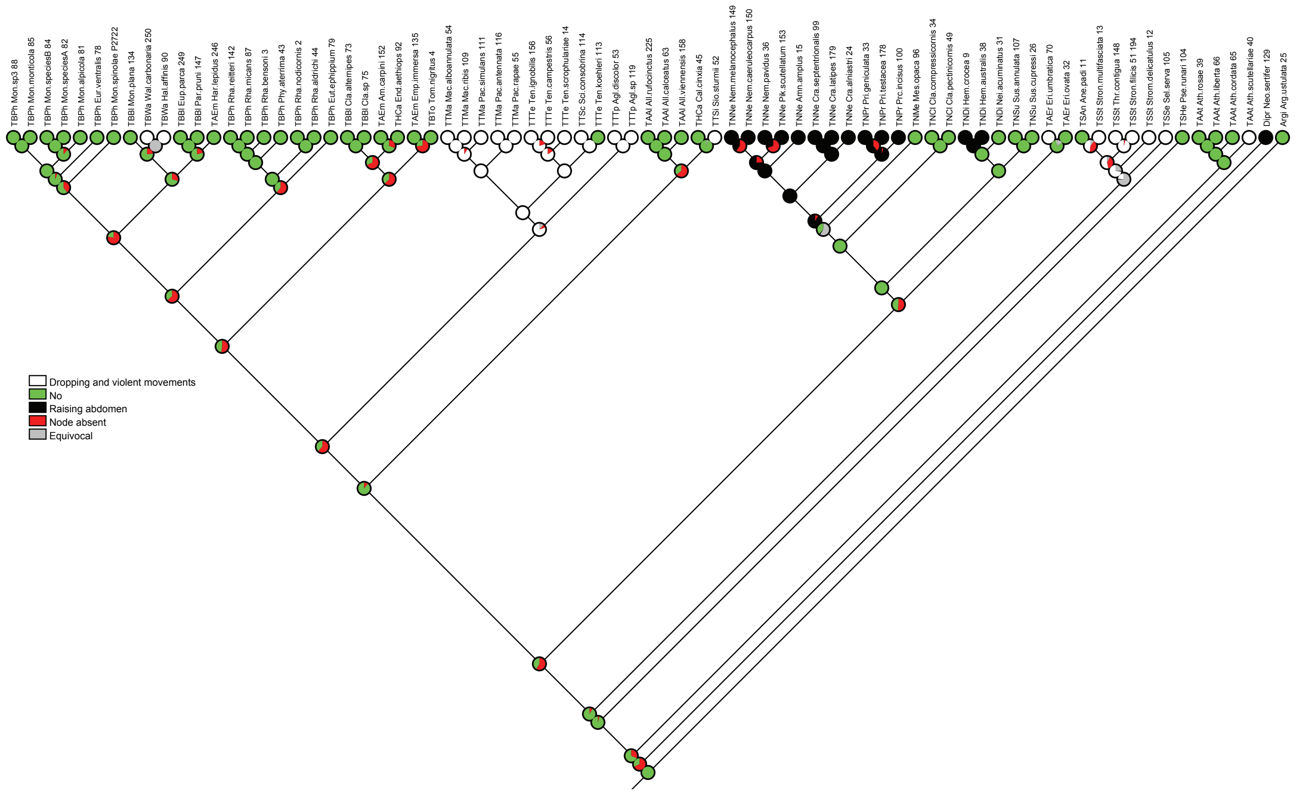

H. Predominant body coloration

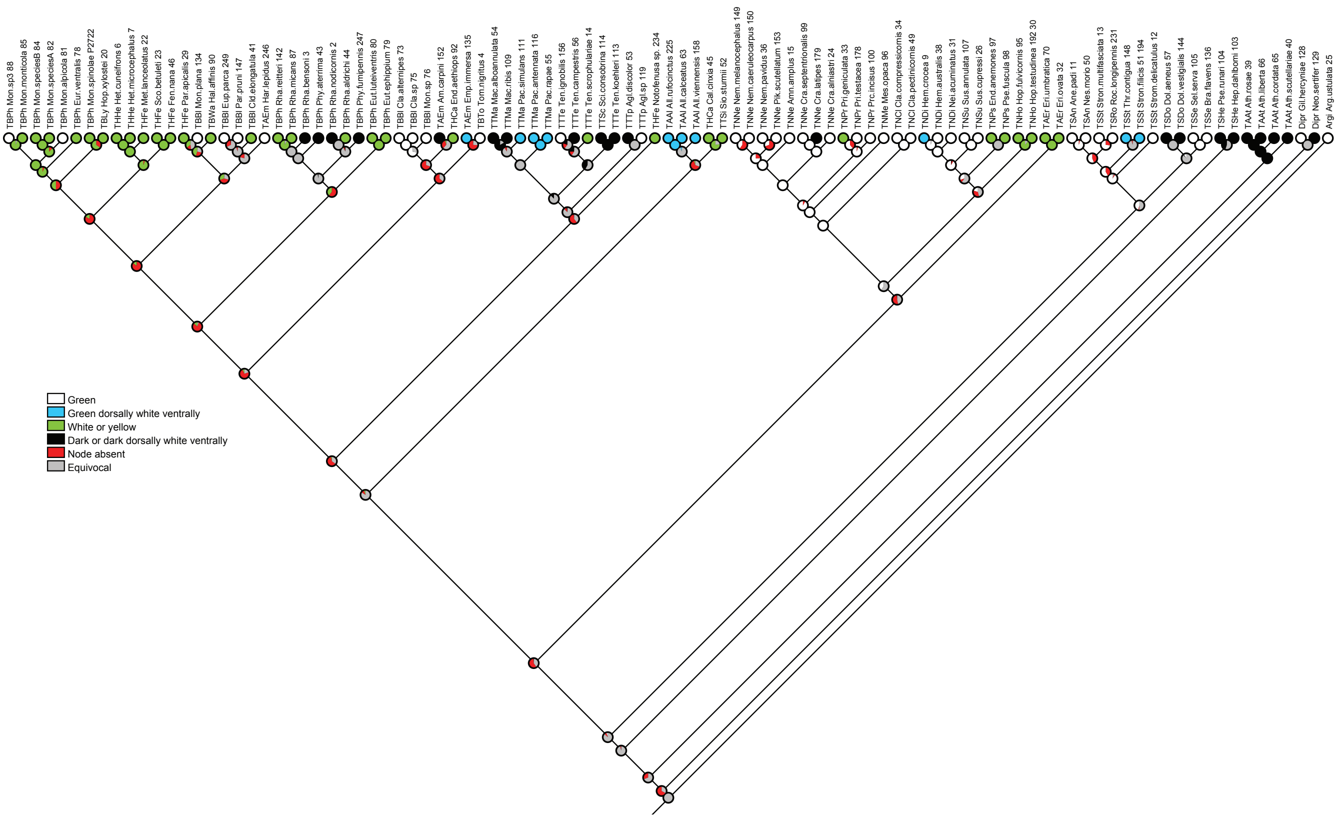

I. Distinct dark to black spots

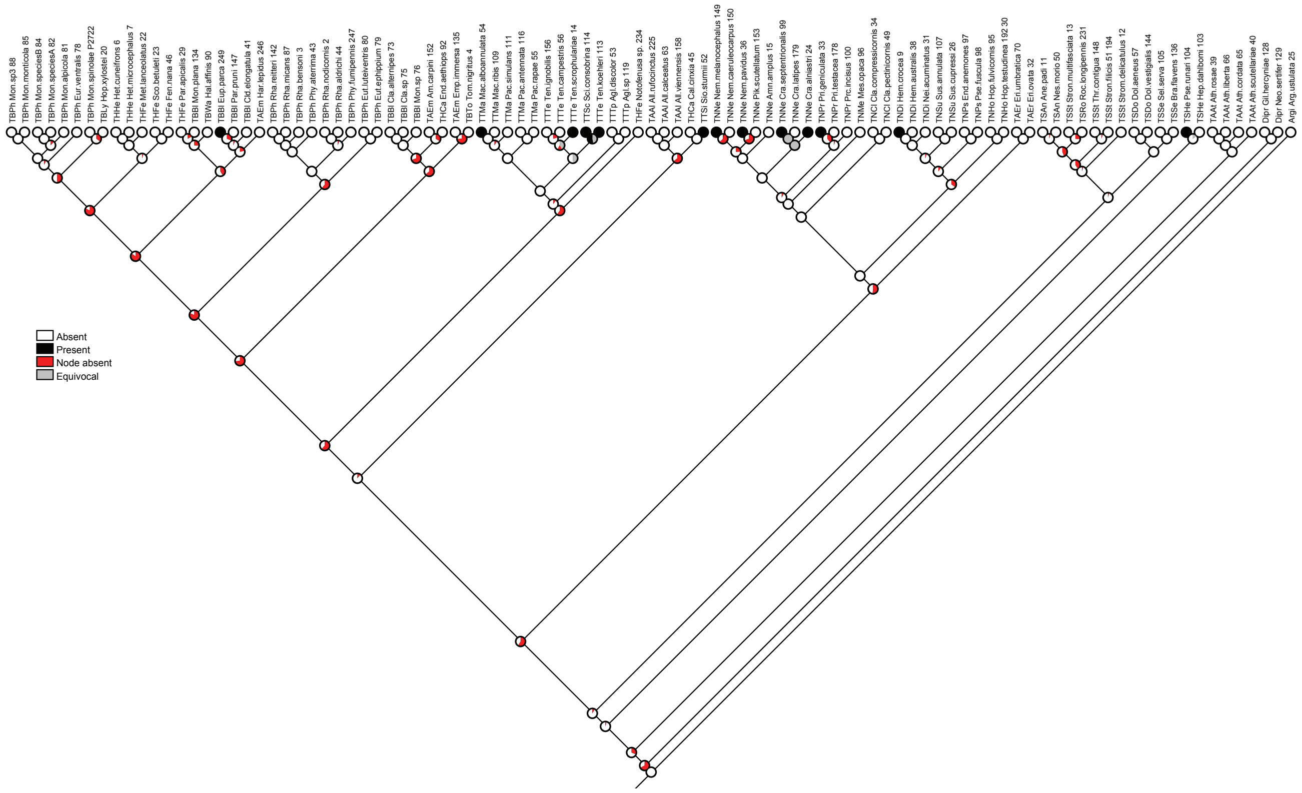

J. Exocrine ventral glands

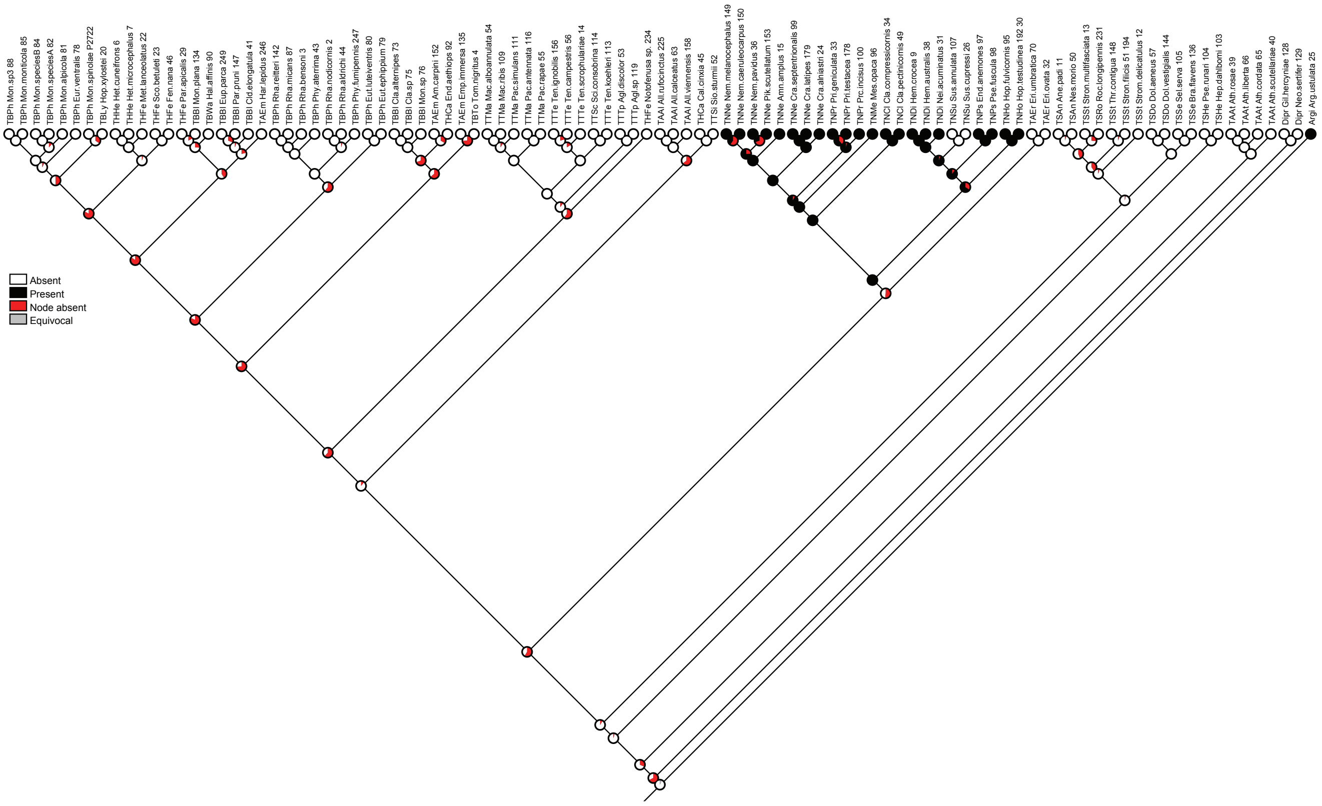

K. Body setation and protrusions

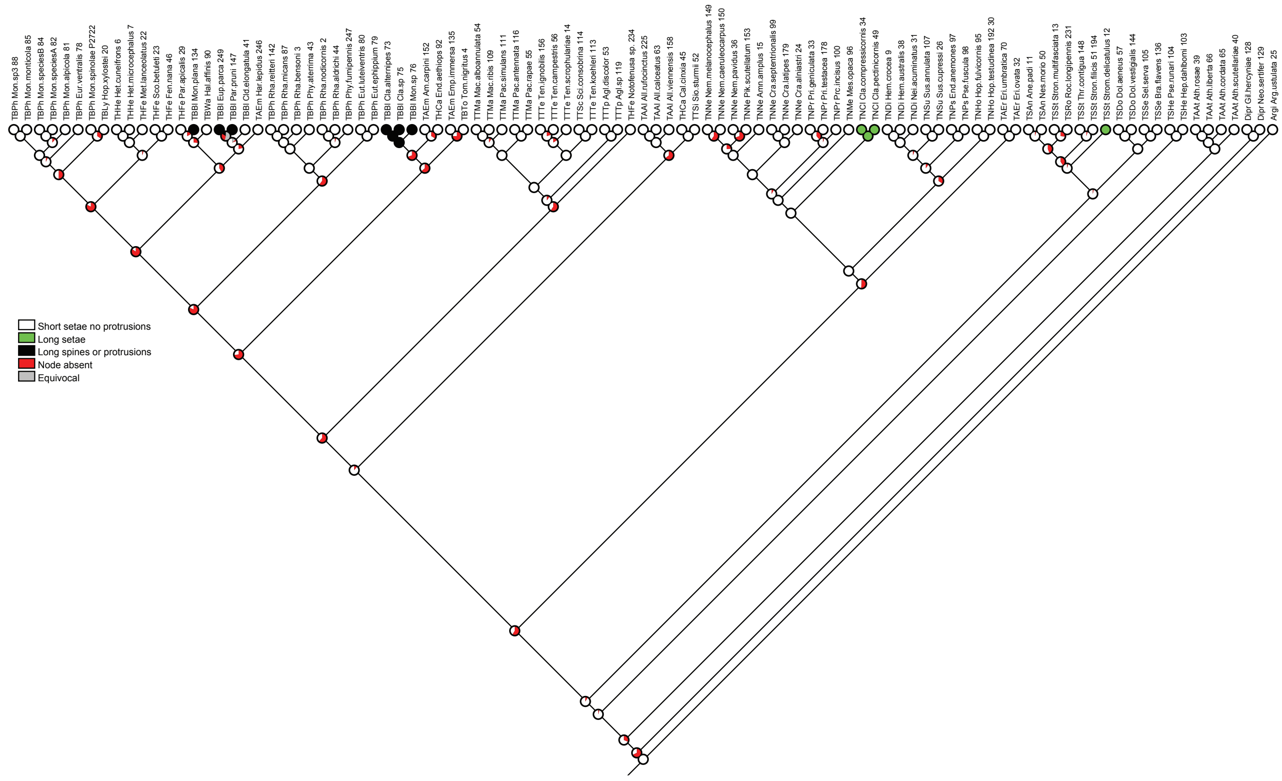

L. Integumental wax layer

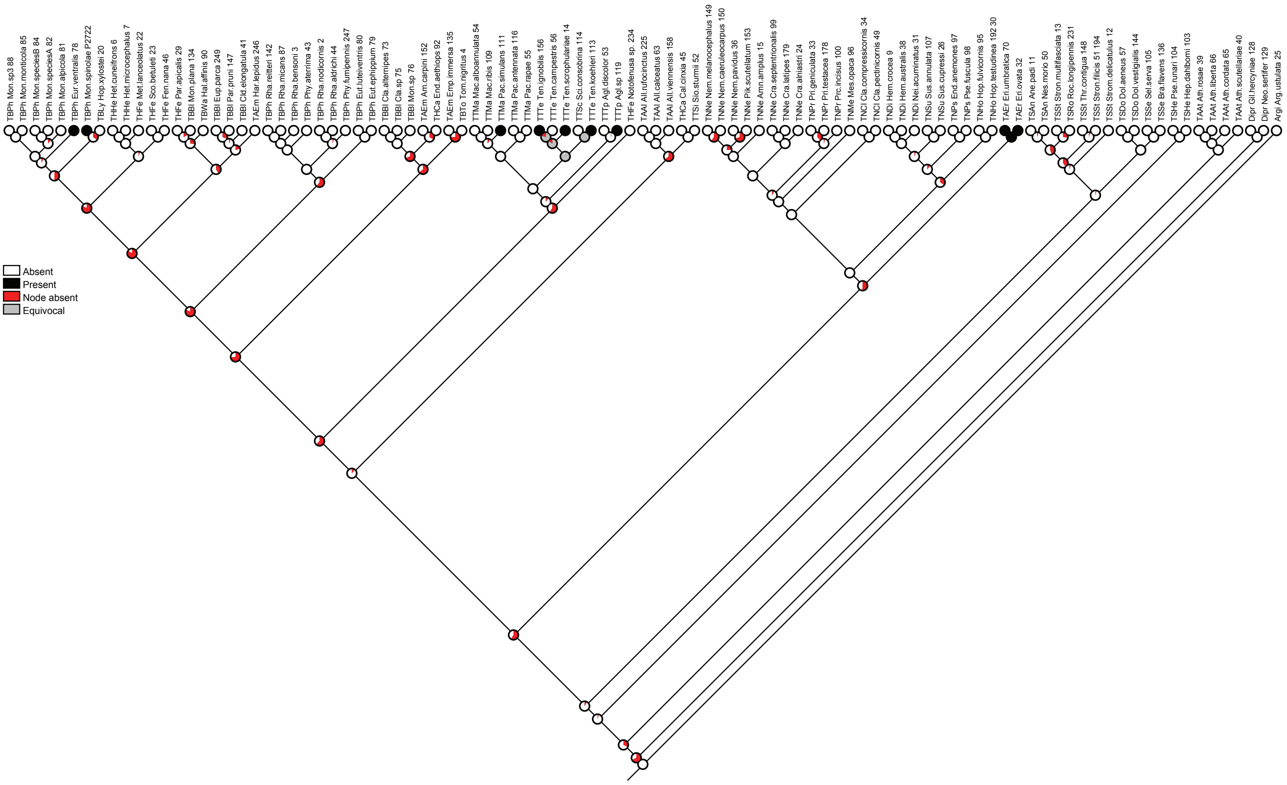

M. Easy bleeding

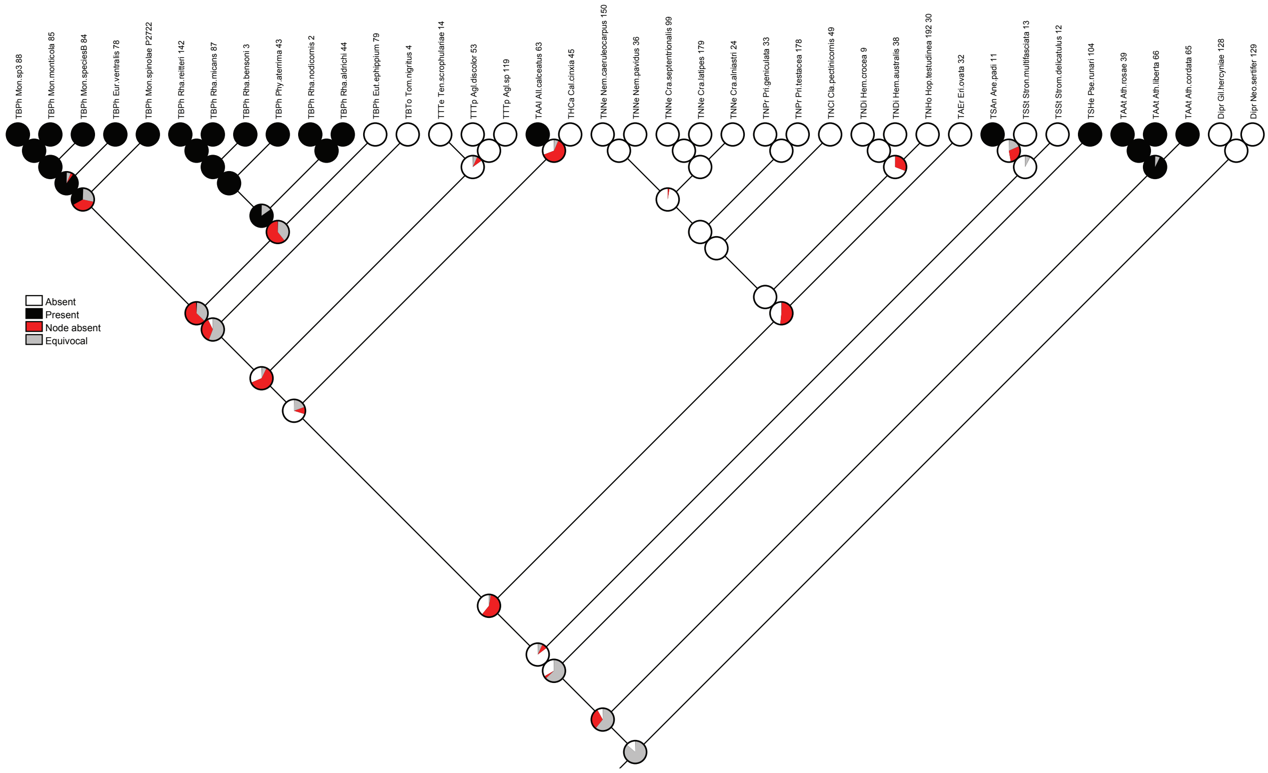

Supplement: Additional file 4 — Reconstruction of ancestral states in host plant associations as well as ecological and defensive traits (A–M) based on maximum-likelihood optimization across a sample of 1,000 post-burnin trees from the Dataset 2 BEAST analysis. Results are summarized across the MCC topology (Figure 3), pie charts on nodes show proportions of trees with uniquely best states with the decision threshold set to T = 2. [file 1471-2148-13-198-S4.pdf]
